# Combined effects of quinic acid and isochlorogenic acid B on LPS-induced inflammation and pyroptosis in MAC-T cells and mouse mammary glands

**DOI:** 10.3389/fvets.2025.1476302

**Published:** 2025-06-05

**Authors:** Xiang Lv, Quan Long, Yuchen Kang, Tao Lin, Caode Jiang

**Affiliations:** ^1^College of Animal Science and Technology, Southwest University, Chongqing, China; ^2^School of Life Science, Leshan Normal University, Leshan, China

**Keywords:** quinic acid, isochlorogenic acid B, mastitis, NF-κB, NLRP3

## Abstract

**Introduction:**

Quinic acid (QA) and isochlorogenic acid B (ICAB) have been demonstrated to possess antioxidant, anticancer, and anti-inflammatory properties. However, their combined efficacy in protection against mastitis requires further investigation. This study aims to examine the inhibitory effects and mechanisms of QA and ICAB combination on lipopolysaccharide (LPS)-induced inflammation and pyroptosis in bovine mammary epithelial cells (MAC-T) and mouse mammary tissue.

**Methods:**

The optimal concentrations of QA and ICAB for treating MAC-T cells were determined using Cell Counting Kit-8 (CCK-8). Expression levels of inflammatory factors, oxidative stress and pyroptosis indicators were assessed by ELISA. Immunohistochemistry was employed to detect CD3 levels in mouse mammary glands, while Western blot and immunofluorescence techniques were used to measure the expression levels of NOD-like receptor 3 (NLRP3) inflammasome, caspase-11, gasdermin D (GSDMD), and p65 of nuclear factor κB (NF-κB) in MAC-T cells.

**Results:**

QA (60 μg/mL) and ICAB (20 μg/mL) co-treatment significantly enhanced MAC-T cell activity (*p* < 0.05). Combined treatment of QA and ICAB significantly decreased the expression of LPS-induced inflammatory factors (TNF-*α*, IL-1β, and IL-6) and oxidative stress factors (COX-2 and iNOS) in both MAC-T cells and mouse mammary glands (*p* < 0.05), and dose-dependently lowered the levels of pyroptosis indicators (ROS, LDH, and IL-18) (*p* < 0.05). After intraperitoneal injection of QA (20 mg/kg) and ICAB (5 mg/kg), LPS-treated mice exhibited significantly reduced expression of CD3 (*p* < 0.05) and decreased T lymphocyte infiltration in the mammary gland, with a pronounced effect compared to QA and ICAB treatment alone. The combined administration of QA and ICAB also effectively suppressed the expression levels of NF-κB (IκBα, p65, p-IκBα, and p-p65) induced by LPS in MAC-T cells and mouse mammary glands, as well as the nuclear translocation of NF-κB in MAC-T cells (*p* < 0.05). Furthermore, the protein levels of NLRP3 inflammasome (NLRP3, ASC, and caspase-1), caspase-11, and GSDMD were significantly reduced (*p* < 0.05) compared to QA and ICAB treatment alone.

**Conclusion:**

QA and ICAB synergistically inhibits the inflammatory response and pyroptosis in MAC-T cells and mouse mammary glands through modulation of NF-κB pathway and NLRP3 inflammasome. This study contributes novel insights into combination of QA and ICAB in the prevention and treatment of mastitis.

## Introduction

Mastitis is a prevalent infectious disease in the cattle industry, known for its high incidence rate and costly prevention and treatment. It significantly impacts the health of dairy cows and the safety of dairy products (1). *Escherichia coli* (*E. coli*) is the primary pathogenic bacteria responsible for clinical acute mastitis due to the fact that lipopolysaccharide (LPS) in the outer membrane of *E. coli* triggers inflammatory and pyroptotic responses through the nuclear factor kappa B (NF-κB) signaling and NOD-like receptor 3 (NLRP3) inflammasome (2).

Pyroptosis, an inflammatory form of programmed cell death, is regulated by caspase-1 and inflammatory factors (3). In the LPS-induced pyroptosis signaling pathway, LPS binds to Toll-like receptor 4 (TLR4) dimers and activates NF-κB and the NLRP3 inflammasome, which in turn triggers the formation of the N terminus of gasdermin-D (GSDMD) protein (GSDMD-N) and caspase-1 to cause cell membrane perforation and the release of lactate dehydrogenase (LDH) and proinflammatory factors like IL-1β and IL-18, thereby exacerbating inflammatory response (2, 4).

Antibiotics is primarily recommended for mastitis treatment in dairy cows; however, as a result of drug resistance in pathogenic bacteria, antibiotic therapy has been frequently proven to be ineffectual, resulting in persistent bovine mastitis risks to consumer health (5). Consequently, alternatives to antibiotics have been obtained wide attention with emphasis on bioactive compounds derived from plants due to their beneficial advantages of antibacterial, anti-inflammation, and low toxicity (6, 7). Actually, bioactive compounds like alkaloids, flavonoids, essential oils, and saponins have been investigated to treat inflammation, cancer, and neurological diseases (8, 9). Particularly, a lot of researchers have reported synergistic effects of plant components on inflammation in animals and humans (10). For examples, previous studies have demonstrated that curcumin, polydatin, and quercetin synergistically counteract pro-oxidative and proinflammatory signals induced by both senescence and hyperglycemic conditions (11). Furthermore, paeoniflorin and baicalin combination has demonstrated greater efficacy in reducing oral inflammation (12), while astragaloside IV and tanshinone combination has been found to significantly decrease cardiomyocyte inflammation (13). Hence, plant components and their combination provide prophylactic and therapeutic purposes on mastitis.

Recently, we reported the anti-inflammatory effect of the ethanol extract of *Rhapontici Radix* in LPS-induced MAC-T cells and mouse mammary glands, and isochlorogenic acid B (ICAB) and quinic acid (QA) were two of the main components in the extract (14). In nature, ICAB is a dietary flavonoid also found in plants such as honeysuckle, *Gynura procumbens*, and Hainan white leaves (15, 16), while QA is a phenolic acid that is widely distributed in microorganisms and plants (17). Accumulating evidence show that both ICAB and QA has anti-informatory, antioxidant, and anti-cancer properties, etc. (18–21). For examples, ICAB offers protective effect against liver fibrosis in mice with non-alcoholic steatohepatitis by inhibiting oxidative stress through the pathway of nuclear factor erythroid-2-related factor 2 (18), and it reduces ear swelling from dermatitis and chronic itching, as well as lead-induced anxiety, depression, and neuroinflammation by modulating the brain-derived neurotrophic factor signaling pathway in mice (19). Further reports have documented that QA decreases mRNA levels of PPAR-*γ*-2, TNF-*α*, IL-1β, and IL-6 in the adipose tissue of mice with hyperlipidemia; dietary supplementation of QA facilitated gastrointestinal tryptophan and nicotinamide synthesis and DNA repair, and suppresses the expression of NF-κB caused by AlCl_3_ (22). Additional research has shown that QA from *Lonicerae Japonicae Flos* and honeysuckle hinders biofilm formation of *Pseudomonas aeruginosa* and is widely utilized in the synthesis of drugs with antioxidant and antibacterial properties (21). While recent work have revealed the anti-mastitis effect of ICAB (14) and inhibitory effect of QA on inflammation induced by high fat diet in mice (22), whether they have a synergistic impact on mastitis remains unclear.

Therefore, in this study, LPS-induced bovine mammary epithelial MAC-T cells and mouse mammary tissue were used to establish the *in vitro* and *in vivo* inflammation models, respectively. The combined effects of QA and ICAB on the expression of inflammatory factors and activation of the NF-κB pathway and NLRP3 inflammasome were also investigated.

## Materials and methods

### Cell culture and treatment

MAC-T cells (BNCC, China) were cultured in DMEM basal medium (Gibco, USA) supplemented with 10% FBS (Gibco, USA), 100 U/mL penicillin, and 1% streptomycin (Solarbio, China). The cells were maintained in a 5% CO_2_ and 37°C incubator (Forma Series 3 WJ, Thermo, USA). Lactation condition of MAC-T cells was induced according to our recent work by a combination of 1 μg/mL hydrocortisone, 5 μg/mL prolactin, and 5 μg/mL insulin (14). The cells, reaching 90% confluence in a logarithmic growth phase, were treated with 1 μg/mL LPS (L8880, Solarbio, China) combined with QA (60 μg/mL), ICAB (20 μg/mL), and QA (60 μg/mL) + ICAB (20 μg/mL) (14, 23) in five separate experiments for 24 h. Cells in culture medium without LPS, QA, and ICAB were used as control group, while cells in LPS without QA and ICAB were used as inflammatory model group.

### CCK-8 toxicity test

MAC-T cells (100 μL cell suspension) were transferred to each well of 96-well plates at a density of 2 × 10^6^ cells in five replicates. For optimal concentration of QA and ICAB, the above cells were exposed to the treatment of QA (purity >98.0%, Soleba, China) or ICAB (purity > 98.0%, Soleba, China) at 0, 5, 10, 20, 40, 60, 80, and 100 μg/mL in five replicates (14). Subsequently, 60 μg/mL QA, 20 μg/mL ICAB, and QA (60 μg/mL) + ICAB (20 μg/mL) were administrated in 5 replicates, and 100 μL of PBS buffer was added around the treatment wells. After 24 h of treatment, 10 μL of CCK-8 solution (Soleba, China) was added to each well, followed by an incubation of 2 h. The optical density value (OD) at 450 nm was measured using SpectraMax iD3 (Moleculer Devices, Shanghai, China).

### Animal experiments

The animal procedures were approved by the Local Ethics Committee on Animal Experiments of Southwest University, China (No. IACUC-20230904-02). Forty-five 6-week-old BALB/c mice, consisting of 15 male mice and 40 female mice, were procured from Enbi Biotechnology (Shanghai, China). The female mice were housed in pairs per cage, while each male mouse was housed in a cage. The mice were provided with water and chew diet (Byrness Weil biotech Ltd., Chongqing, China) *ad libitum* and maintained under a 12 h light/dark cycle. After a period of 7 days, two female mice and one male mouse were co-housed in a cage for breeding. Seven days postpartum, the female mice were randomly assigned to one of the five groups: the control group, LPS group, LPS + QA (20 mg/kg) (24), LPS + ICAB (5 mg/kg) (25), and LPS + QA (20 mg/kg) + ICAB (5 mg/kg) group. Each group consisted of 5 replicates. QA and ICAB were dissolved in PBS and injected intraperitoneally, while the control and LPS groups were injected intraperitoneally with an equal volume of PBS. One hour post-injection, the mice were anesthetized with 2% sodium pentobarbital (50 mg/kg) (14), and LPS (200 μg/mL in 50 μL of PBS) was administrated with 32-gauge needles into the mammary ducts of the fourth pair of nipples in the female mice. Twenty-four hours after LPS injection, when the mammary gland was red, swollen, and hyperemia, 5 mL blood was collected from the eyeball and injected into a 10 mL centrifuge tube containing 0.1 mL heparin sodium (10 mg/mL saline solution). After centrifugation at 3000 g and 4°C for 30 min, the serum was collected. Then, the mice were euthanized via cervical dislocation, and the collected mammary tissues were stored at −80°C until use.

### ELISA assay

The cells treated as above were planted in 6-well plates or 12-well plates. The levels of IL-1β, IL-18, TNF-*α*, IL-6, iNOS, and COX-2 in the cell supernatant and mouse serum were determined using ELISA kits (Solebao, Beijing) according to the manufacture’s instruction. The OD_450_ values were measured in xMark^™^ (BIO-RAD, CA, United States).

### ROS and LDH assay

Supernatant of cells and serum of mice treated as described above were collected. The activity of LDH and content of ROS were measured at 460, 525 nm, and 450 nm, respectively, using LDH and ROS assay kits (Quanzhou Risin Biotechnology Co., LTD.). Each assay was performed in triplicate.

### Western blotting

The treated cells and mouse L4 tissues were ground. Total protein was extracted using the total cell protein extraction kit (Solarbio, Beijing) and quantified using the BCA Protein Quantification Kit (Solarbio, Beijing). Western blotting was carried out according to our recent method (14, 23) in triplicate, with *β*-actin as an internal control. The primary antibodies used included β-actin (bsm-33036 M), p65 (bs-23217R), phosphorylated p65 (p-p65, bs-0982R), IκB (bs-1287R0), p-IκB (bs-2513R), ASC (10500-1-AP), NLRP3 (19771-1-AP), caspase-1 (22915-1-AP), GSDMD (20770-1-AP), and caspase-11 (21741-1-AP) (Proteintech, Wuhan, Hubei) at a dilution ratio of 1:1,000. The secondary antibody included Goat Anti-rabbit IgG/HRP (BS13278; Bioss, Beijing) at a dilution ratio of 1:5,000. The target band was visualized using an ECL chemiluminescence system (Gel Doc XR, Bio-Rad, USA) and quantitatively analyzed using Image Lab (version 8.2, Bio-Rad, USA).

### Detection of p-p65 nuclear transfer by immunofluorescence

MAC-T cells were cultured in 96-well plates at 5,000/well. For immunofluorescence, the cells were incubated with p-p65 antibody, followed by incubation with Goat Anti-rabbit IgG/Cy3 secondary antibody. Nuclear staining was performed using 4′,6-diamino-2-phenyllindol (DAPI) and observed using a fluorescence inverted microscope (Leica, Wetzlar, Germany). For ELISA test of p-p65 nuclear transfer, nuclear protein of the cultured cells was extracted using the Nuclear Protein Extraction Kit and quantified using the BCA Protein Quantification Kit (Solebao, Beijing, China) (14, 23). The levels of p-p65 in the nuclei of MAC-T cells were determined using the p65 ELISA Kit (Solebao, Beijing, China) in triplicate.

### Immunohistochemical analysis (IHC)

Formaldehyde fixation, slicing, and *in situ* detection of antibody and antigen binding were outlined in our previous reports (14, 23). Briefly, slices of mouse R4 mammary glands were incubated with the primary antibody against CD3 (1:150; Abcam, Shanghai, China) overnight at 4°C. Then, the slices were incubated with secondary antibody [i.e., goat anti-rabbit IgG (H + L)-HRP (BS13278)] (1:500; Bioss, Beijing, China) for 1 h at room temperature, and were visualized with DAB. The nuclei were counterstained with hematoxylin, and IgG instead of the primary antibody was used as a negative control.

### Statistical analysis

All results were represented as mean ± standard error of the mean. The data were analyzed by one-way ANOVA using v26.0 Statistical Product Service Solutions (SPSS) software, followed by the Tukey *post hoc* test. Statistical significance was set at *p* < 0.05 or *p* < 0.01.

## Results

### Effect of QA + ICAB on MAC-T viability

**Figure 1:**
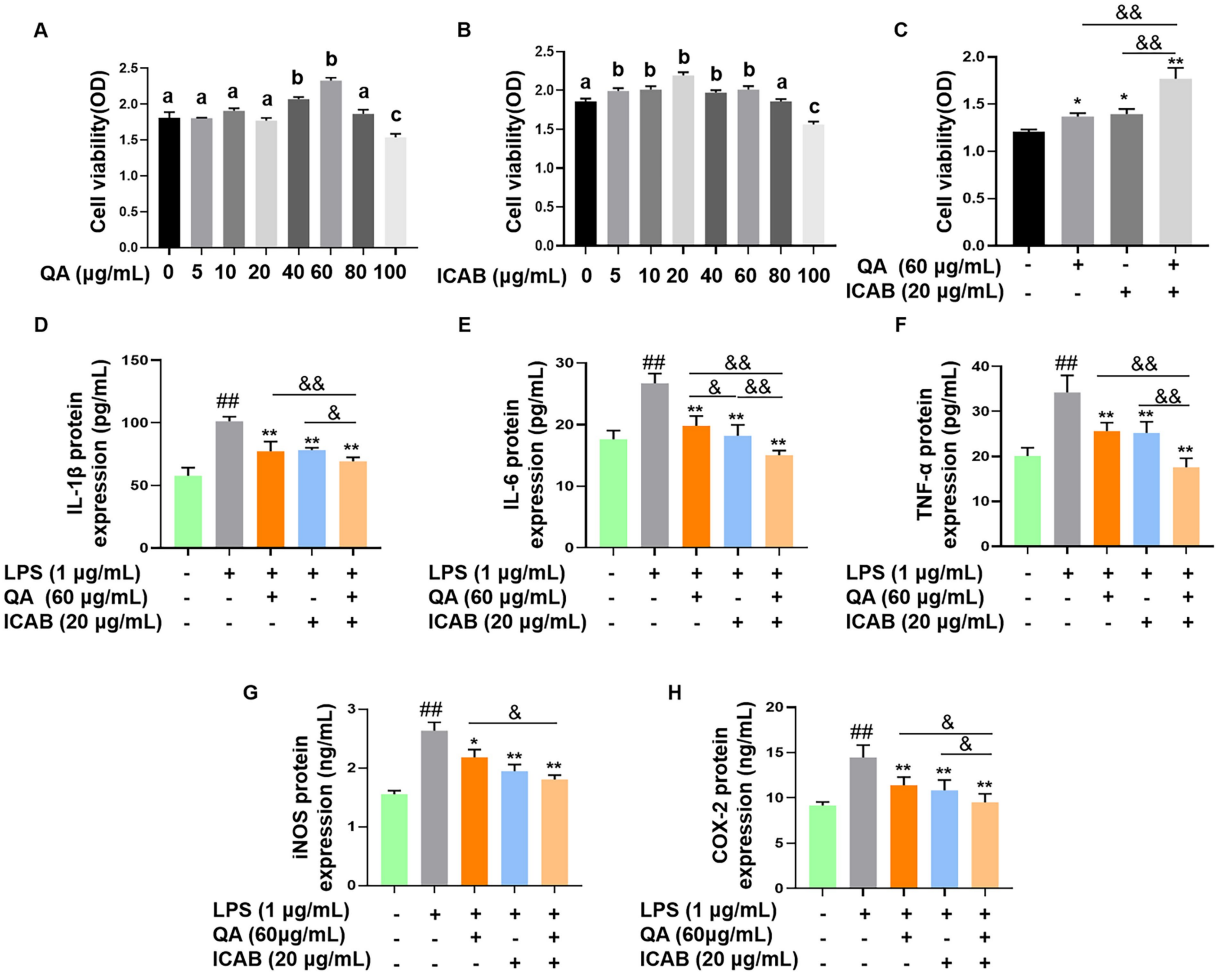
Cell viability test and inhibitory effects of QA and ICAB co-treatment in MAC-T cells. Cell viability was measured by CCK-8 assay after MAC-T cells were treated with different concentrations of QA **(A)** and ICAB **(B)**, and co-treated with QA and ICAB **(C)** for 24 h. ELISA was utilized to measure the protein levels of IL-1β (D), IL-6 **(E)**, and TNF-*α* **(F)**, as well as the activities of iNOS **(G)** and COX-2 **(H)**. Values represent means ± SEM (n = 5). Above bars, the same letters mean *p* > 0.05, different letters mean *p* < 0.05. ## indicates *p* < 0.01 vs. the blank controls, while * and ** indicate *p* < 0.05 and *p* < 0.01 vs. the LPS treatments, respectively. For multiple comparisons, & *p* < 0.05 and && *p* < 0.01.

MAC-T cells were treated with different concentrations of QA or ICAB to screen for their optimal treatment concentrations. The results of the CCK-8 assay demonstrated that the treatment of QA at 60 μg/mL or ICAB at 20 μg/mL had the highest cell viability, which was significantly higher than that of the control group (Figures 1A,B, *P* < 0.05). Importantly, the treatment of QA (60 μg/mL) + ICAB (20 μg/mL) exhibited a greater enhancement in cell viability compared to QA or ICAB treatment alone (Figure 1C, *P* < 0.01).

### Anti-inflammatory effect of QA + ICAB in MAC-T cells

ELISA was employed to assess the expression levels of proinflammatory factors in LPS-induced MAC-T cells. As expected, both QA or ICAB treatment significantly reduced LPS-induced expression of TNF-*α*, IL-1β, IL-6, iNOS, and COX-2 (Figures 1D–H, *p* < 0.01). Moreover, QA + ICAB treatment significantly lowered the expression levels of TNF-α, IL-1β, IL-6, and COX-2 compared to the treatments of QA or ICAB alone (*p* < 0.05). However, iNOS expression level of QA + ICAB group was not significantly different compared with ICAB group (*p* > 0.05).

### Regulation of NF-κB inflammatory pathway by QA + ICAB in MAC-T cells

**Figure 2:**
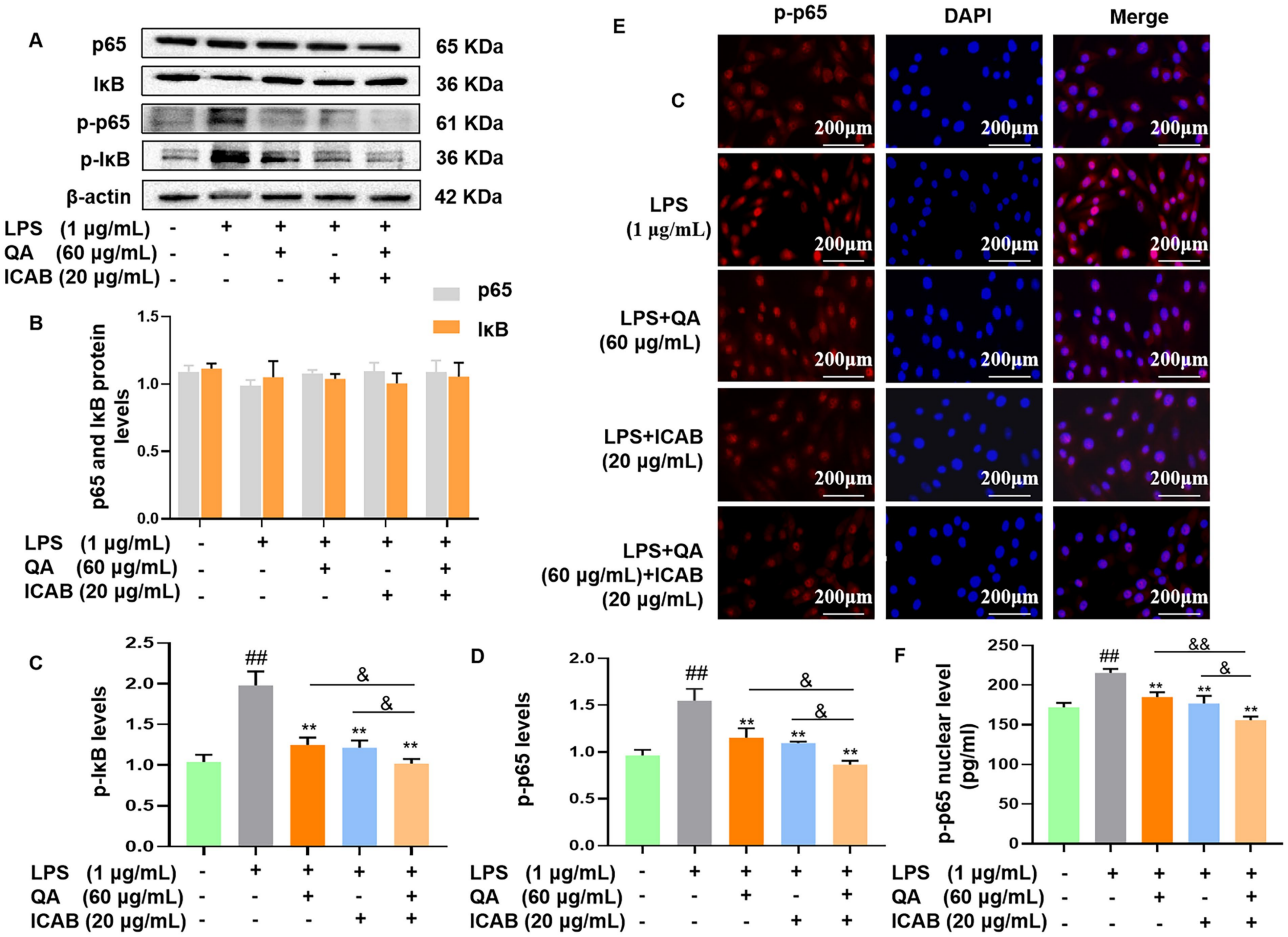
Western blot detection and immunofluorescence analysis of NF-κB activity in MAC-T cells. Cells were incubated with LPS and co-treated with QA and ICAB individually or jointly for 24 h. Western blot were analyzed for p-p65/p65 and p-IκB/IκB **(A)**. Protein levels of p65 and IκB **(B)**, p-IκB **(C)** and p-p65 **(D)** were relative to β-actin. For immunofluorescence analysis **(E)**, p-p65 was labeled with fluorescence Cy3 (red), while nucleus was marked with fluorescence DAPI (blue). ELISA was used to test the levels of p-p65 in the nuclear **(F)**. Values represent means ± SEM (n = 3). ^##^ indicates *p* < 0.01 vs. the blank control, while * and ** indicate *p* < 0.05 and *p* < 0.01 vs. the LPS treatment, respectively. For multiple comparisons, & *p* < 0.05 and && *p* < 0.01.

We assessed protein and phosphorylation levels of the key components of NF-κB (p65 and IκB) in LPS-stimulated MAC-T cells. Our results indicated no significant difference in p65 and IκB protein expression levels among treatment groups (Figures 2A,B, *P* > 0.05). However, treatment with QA, ICAB, and QA + ICAB markedly decreased LPS-induced p-p65 and p-IκB levels (Figures 2C,D, *P* < 0.01). Especially, combined treatment of QA and ICAB showed superior efficacy compared to QA or ICAB treatment alone (Figures 2C,D, *P* < 0.05).

We further examined nuclear translocation of p-p65 in MAC-T cells by immunofluorescence. As expected, the results showed that LPS enhanced the fluorescence signal of nuclear p-p65, while QA, ICAB, and QA + ICAB treatments decreased LPS-enhanced fluorescence signal of nuclear p-p65 (Figure 2E). As our previous work proved the efficacy of the Nuclear Protein Extraction Kit in fractioning of nuclear protein (23), nuclear content of p-p65 was measured. In consistence with immunofluorescence detection, the nuclear fraction of p-p65 in QA + ICAB combination was significantly lower than that of QA (*p* < 0.01) or ICAB (*p* < 0.05) treatment alone.

### Inhibitory effects of QA + ICAB on the indicators and pathway of pyroptosis in MAC-T cells

**Figure 3:**
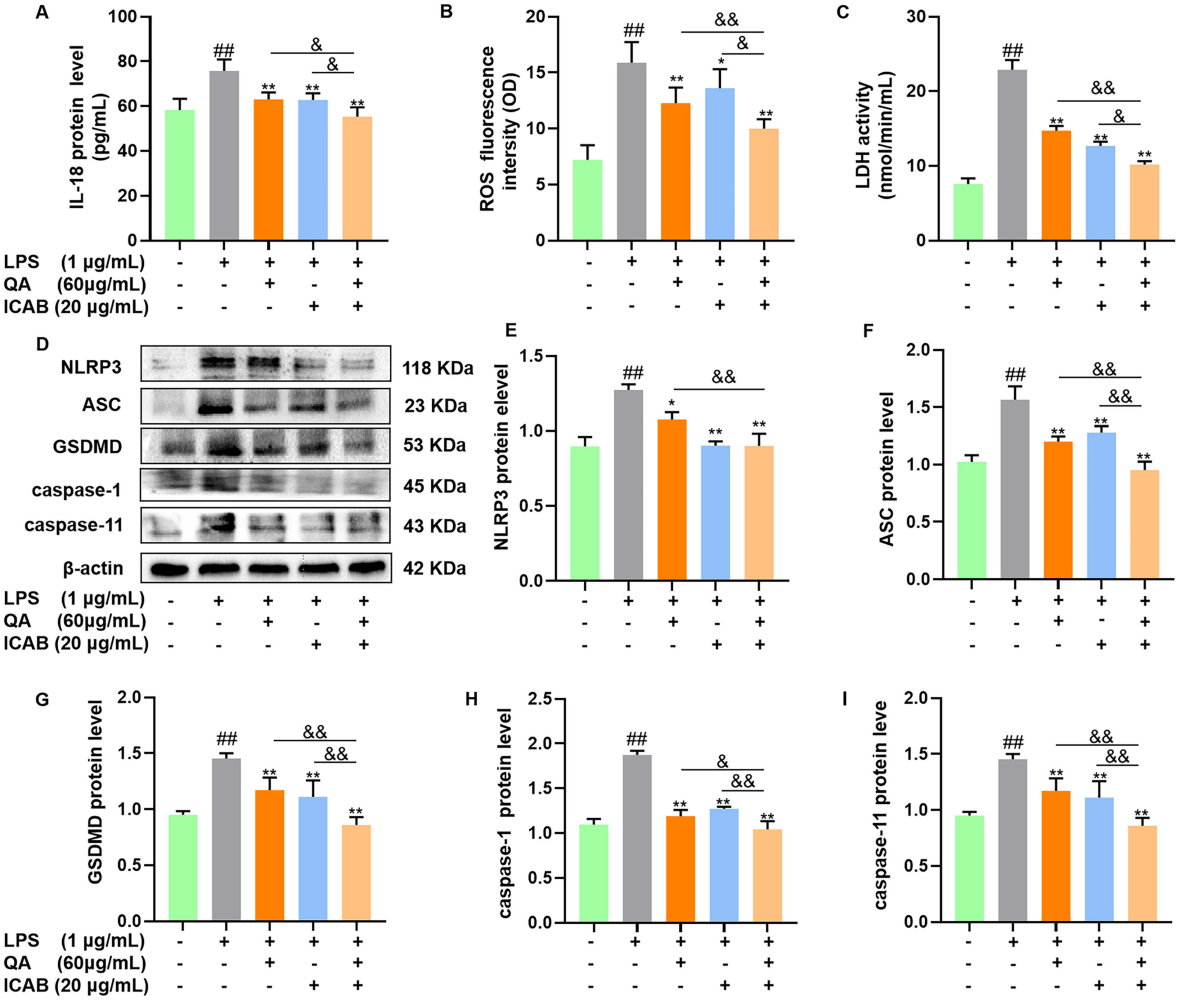
Effect of QA and ICAB combination on pyroptosis in MAC-T cells. Cells were treated with QA, ICAB, QA + ICAB. Levels of IL-18 **(A)**, ROS **(B)**, and LDH **(C)** were measured using ELISA or commercial kits. Western blot **(D)** were used to detect the protein levels of NLRP3 **(E)**, ASC **(F)**, GSDMD **(G)**, caspase-1 **(H)**, and caspase-11 **(I)** relative to β-actin. Values represent means ± SEM (n = 3). ## indicates *p* < 0.01 vs. the blank control, while * and ** indicate *p* < 0.05 and *p* < 0.01 vs. the LPS treatment, respectively. For multiple comparisons, & *p* < 0.05 and && *p* < 0.01.

First, the expression levels of pyroptosis indicators ROS, LDH, and IL-18 were examined. As shown in Figure 3, compared with blank control group, ROS fluorescence intensity, LDH activity, and IL-18 level in MAC-T cells treated with LPS were significantly enhanced (*p* < 0.01); however, QA (*p* < 0.01) or ICAB (*p* < 0.05) treatment significantly reduced levels of the pyroptosis indicators, with more efficacy of their combined treatment (*p* < 0.05).

Next, proteins related to NLRP3-mediated pyroptosis pathway were detected. The Western blot and quantitation results demonstrated that QA (*p* < 0.05) or ICAB (*p* < 0.01) significantly reduced LPS-induced levels of NLRP3, ASC, GSDMD, caspase-1, and caspase-11 (*p* < 0.05), and QA + ICAB combination had better effects than QA or ICAB alone (*p* < 0.05, Figures 3D–J).

### Anti-inflammation and anti-pyroptosis of QA + ICAB in mice

**Figure 4:**
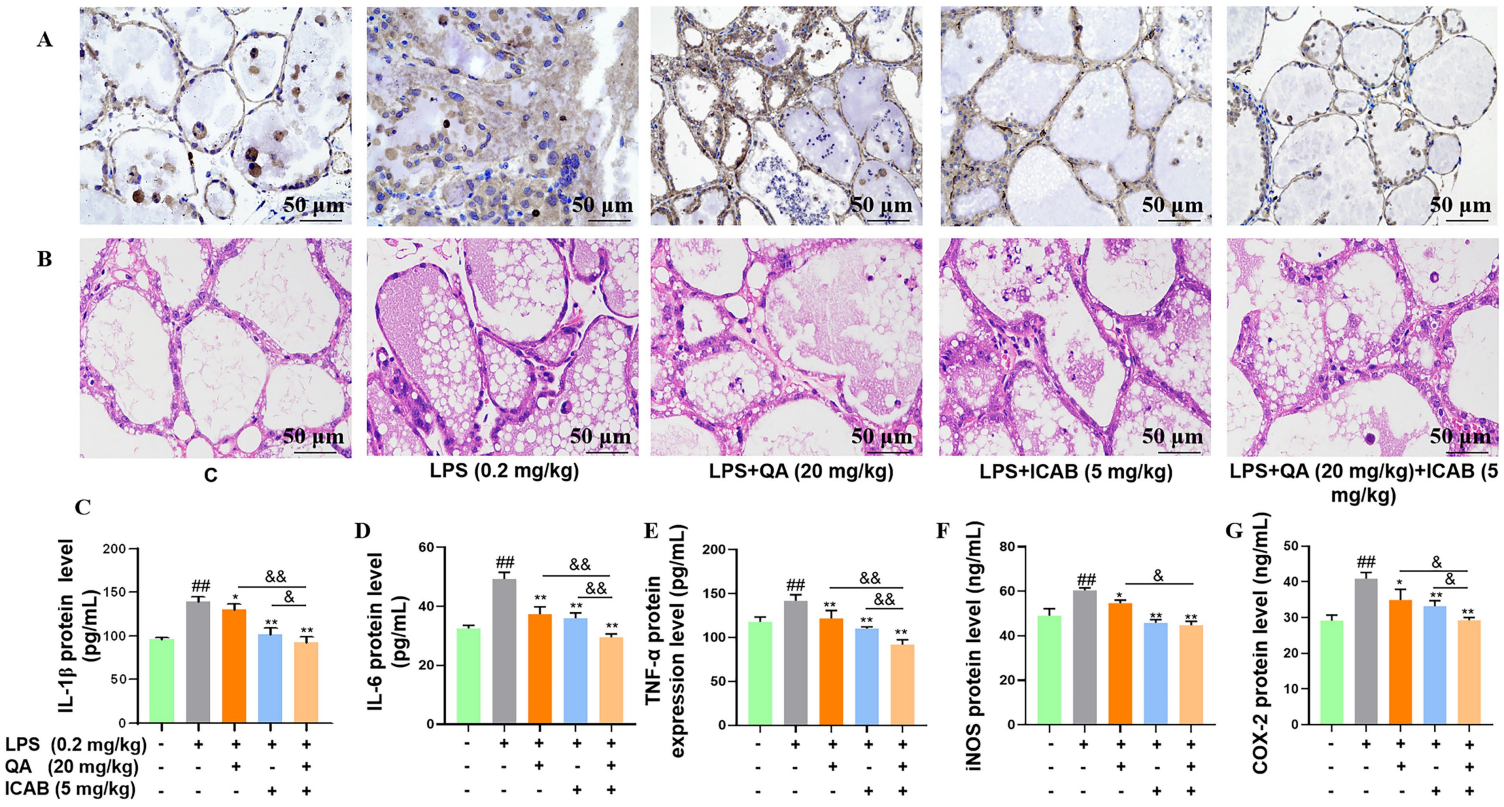
Effect of QA and ICAB co-treatment on LPS-induced inflammation in mouse mammary glands. **(A,B)** Immunohistochemical staining of CD3 and HE staining in the mammary gland. **(C–G)** The protein levels of IL-1β, IL-6, TNF-α, iNOS, and COX-2, which were measured by the ELISA method. Values represent means ± SEM (n = 3). ## indicates *p* < 0.01 vs. the blank control, while * and ** indicate *p* < 0.05 and *p <* 0.01 vs. the LPS treamtments, respectively. For multiple comparisons, & *p* < 0.05 and && *p* < 0.01.

To consolidate our *in vitro* results, we firstly inspected the impacts of QA and ICAB on histopathological changes and inflammation in LPS-administrated mice. IHC results revealed that compared with the control mice, the mammary gland tissue of mice treated with LPS showed a marked increase in CD3, which is a specific molecular marker of T lymphocytes, indicating that leukocyte infiltrated in the lumen; however, the administration of QA, ICAB, and QA + ICAB clearly reversed CD3 staining (Figure 4A). HE staining also unveiled that the administration of QA, ICAB, and QA + ICAB mitigated acinar congestion edema and cellular content release stimulated by LPS (Figure 4B). Especially, QA + ICAB combination significantly lowered the levels of inflammatory factors (TNF-*α*, IL-1β, and IL-6) and oxidative stress factors (COX-2 and iNOS) in mouse mammary glands compared with QA or ICAB treatment alone (*p* < 0.05, Figure 4C).

**Figure 5:**
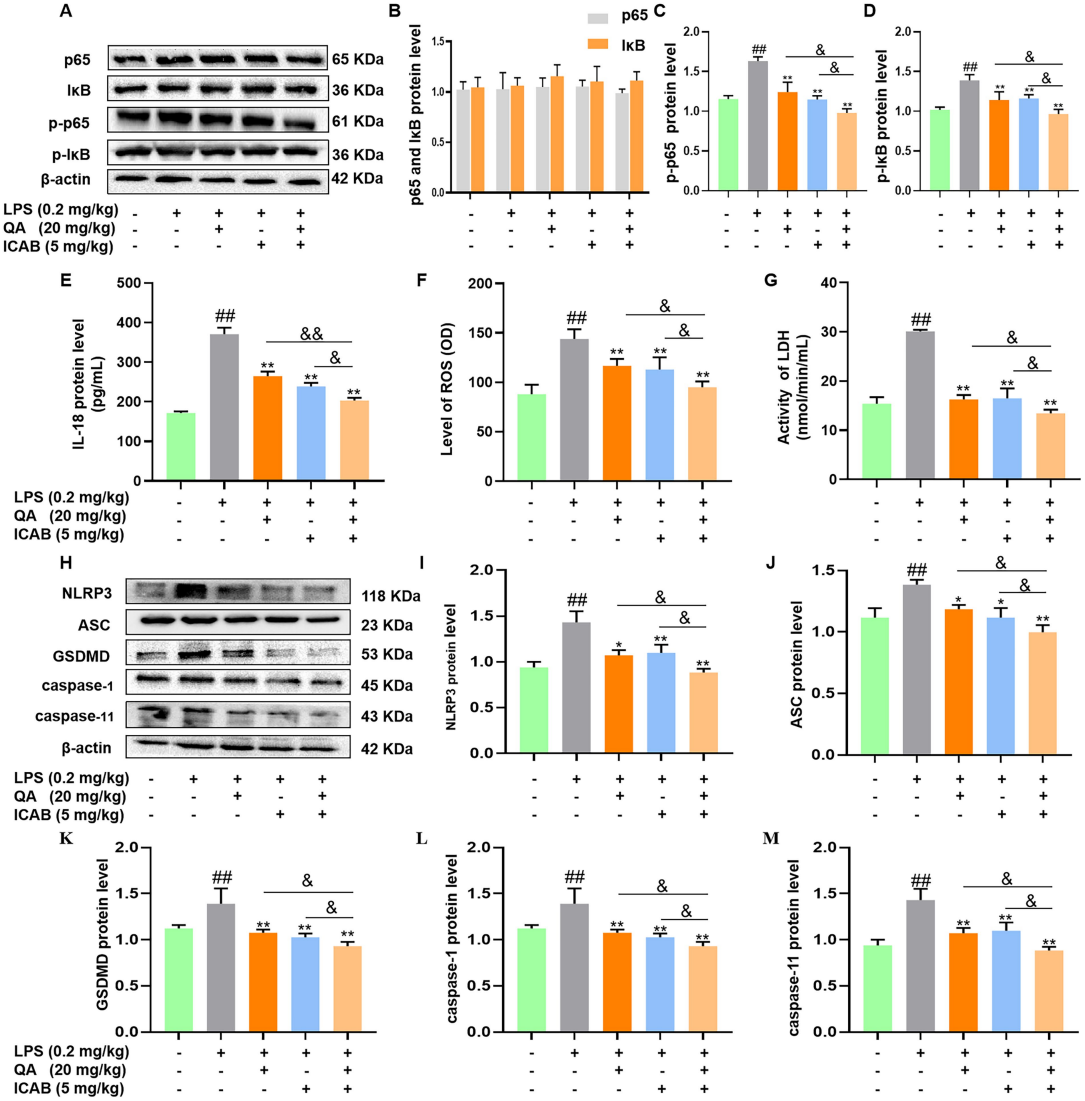
Effects of QA and ICAB co-treatment on NF-κB signaling and pyroptosis in mouse mammary tissue. **(A–D)** Western blot analysis and the protein levels of p65, p-p65, IκB, and p-IκB. **(E–G)** Measurement of IL-18, ROS, and LDH. **(H–M)** Western blot analysis and the protein levels of NLRP3, ASC, GSDMD, caspase-1, and caspase-11. β-actin was used as an internal control. Values represent means ± SEM (*n* = 3). ## indicate *p* < 0.05 and *p* < 0.01 vs. the blank control, while * and ** indicate *p* < 0.05 and *p* < 0.01 vs. the LPS treatments, respectively. For multiple comparisons, & *p* < 0.05 and && *p* < 0.01.

Subsequently, protein and phosphorylation levels of NF-κB components (p65 and IκB) were analyzed in mouse mammary tissue. While differences in p65 and IκB protein levels were not significant among the five treatment groups (Figures 5A,B, *P* > 0.05), QA, ICAB, and QA + ICAB treatments notably reduced LPS-induced p-p65 and p-IκB levels (Figures 5C,D, *P* < 0.05). Particularly, QA + ICAB co-treatment exhibited superior effects compared to QA or ICAB treatment alone (*p* < 0.05).

Finally, the expression of pyroptosis indicators and NLRP3-mediated pathway were measured in mouse mammary tissues. As shown in Figures 5E–G, QA + ICAB co-treatment significantly decreased ROS, LDH, and IL-18 expression levels compared to QA and ICAB treatment, respectively (*p* < 0.05). Additionally, NLRP3, ASC, caspase-1, caspase-11, and GSDMD levels were notably reduced compared to QA or ICAB treatment alone (*p* < 0.05, Figures 5H–M).

## Discussion

Prevention and treatment of mastitis is a focus of dairy industry. Research has reported that lactating mice challenged with pathogens from clinical mastitis cases have similar pathological changes and expression patterns of immune-related genes to those observed in dairy ruminants; MAC-T cells, derived from bovine mammary epithelial cells, have the same biological responses as primary cells (26, 27). To this end, accumulating studies have utilized MAC-T cells and mouse models to evaluate efficacy of new anti-mastitis drugs against *E. coli*, *Staphylococcus aureus,* and other mastitis causing factors (14, 23). According to our reports that LPS triggered an inflammatory response through the NF-κB pathway in MAC-T cells and mouse mammary glands, and LPS induced pyroptosis by activating caspase-11 (28), LPS was applied to stimulate *in vitro* and *in vivo* models in this investigation. We found that LPS administration resulted in elevated levels of proinflammatory factors (TNF-*α*, IL-6, IL-1β, COX-2, and iNOS) both *in vitro* and *in vivo*, and upregulated pyroptosis indicators (ROS, LDH, and IL-18) and histological changes, including T lymphocyte infiltration and acinar congestion edema, in mouse glands (Figure 1, 3, 4), indicating the successful establishment of the *in vitro* and *in vivo* models of inflammation and pyroptosis.

Recent researches reported the effectiveness of ICAB in alleviating lead-induced neuroinflammation in mice and LPS-induced mastitis both in MAC-T cells and mice by reducing the expression of inflammatory cytokines and oxidative indicators (14, 25). Similarly, QA has been shown to lower IL-1β, iNOS, IL-6, and TNF-α expression in a mouse colitis model (29). Actually, QA, a monocaffeoylquinic acid, and ICAB, 3,4-di-O-caffeoylquinic acid, are both chlorogenic acid compounds (17). Structurally, ICAB is characterized by caffeoyl substitution at positions 3 and 4 of QA core, and both phytochemicals exhibiting anti-inflammatory activity suggests their potential synergistic effects (30, 31). Significantly, our study firstly demonstrated that combined treatment of QA and ICAB effectively reduced inflammatory responses in MAC-T cells and mouse mammary glands (Figure 1). Although there is no report on the synergistic effect ICAB and QA, several studies have shown the combination effects of chlorogenic acid and geniposide in alleviating steatohepatitis and of chlorogenic acid, t-resveratrol, and quercetin in improving antioxidant capacity in mice (32, 33). These studies highlight the importance of combined use of plant active ingredients in anti-inflammation and anti-oxidation.

NF-κB is a transcription factor that constitutes a canonical signaling pathway for inflammation and pyroptosis induced by LPS (34). NF-κB, usually composed of p50 and p65, keeps inactive heterodimeric complexes by binding with IκB in the cytoplasm; external stimuli including LPS causes p-IκB and its subsequent ubiquitination degradation, liberating p-p65 into the nucleus to initiate transcription of its target genes (34). Recent studies have demonstrated that QA improves ulcerative colitis in rats by inhibiting TLR4/NF-κB and NF-κB/iNOS/NO signaling pathways (30). Moreover, QA showed an anti-cancer role by promoting the apoptosis of human breast cancer cells (35). Although the effect of ICAB on NF-κB is rarely documented, our *in vivo* and *in vitro* evidence indicated that both QA and ICAB individually reduced LPS-induced p-IκB and p-p65 levels (Figures 2, 3), fully proving the inhibitory effect of QA and ICAB on the NF-κB signaling. The finding that the inhibitory effect of QA and ICAB combination was greater than that of their individual use supports their additive effect on NF-κB signaling.

Pyroptosis and inflammation are intimately linked during mastitis due to microbial infection, endogenous danger signals, and environmental stimuli (2, 4). Pyroptosis can be divided into caspase-1-mediated canonical pathway and caspase-4/5/11 mediated non-canonical pathway; LPS induces pyroptosis through the NLRP3 inflammasome (5). The NLRP3 inflammasome is composed of NLRP3, which recognizes the danger signals and recruits downstream molecules; ASC, which forms the ASC speck as a bridge connecting NLRP3 and caspase-1; and effector caspase-1, which leads to the maturation of proinflammatory cytokines (IL-1β and IL-18) and processes of GSDMD to control pyroptosis and cytokine release. Recent research documented that obtusifolin suppressed LPS-induced inflammation and NLRP3 inflammasome activation in BV2 cells (36); the combinations of fucoxanthin and rosmarinic acid was shown to regulate inflammation by reducing NLRP3, ASC, and Caspase-1 production (37). Although the role of QA and ICAB in the regulation of pyroptosis has rarely been reported, the derivative 3,4,5-tricafeylquinic acid down-regulates the expression of NLRP3 and IL-1β in LPS-stimulated THP-1 macrophages, and inhibits the activation of caspase-1 and the formation of GSDMD-N (11). This study further found that QA and ICAB combination not only inhibited the expression of ROS, LDH, and IL-18 in LPS-induced MACT cells and mouse mammary tissue, but also decreased the protein levels of GSDMD, ASC, NLRP3, caspase-1, and caspase-11 (Figures 3, 4). The superior inhibitory effects of QA and ICAB combination on the proteins related to the NLRP3 inflammasome point to their additive effect on LPS-induced pyroptosis.

Accumulating evidence has strongly indicated that herbal formulas contribute to the multi-target interactions of the complex ingredients of their herbal drugs, thus a lot of researchers have begun to investigate the enhanced therapeutic efficiency of the combined action of bioactive compounds. Mangiferin was identified from the rhizomes of Anemarrhena, while cinnamic acid was identified from the branches of *Cinnamomum cassia*, both serving as representative bioactive compounds of Baihu Guizhi Tang, an anti-rheumatic prescription derived from traditional Chinese medicine; the combination of mangiferin and cinnamic acid exerted a similar clinical efficacy with Baihu-Guizhi decoction in alleviating rat rheumatoid arthritis by targeting TLR4/PI3K/AKT/NF-kB signaling to suppress the activation of the NLRP3 inflammasome and the modulation of GSDMD-mediated pyroptosis (38, 39). QA and ICAB could exert anti-inflammatory effects through distinct targets, and there existed a derivative relationship between them (20, 25). Although the combined inhibitory effects of QA and ICAB on NF-κB and NLRP3 pathways found in this study (Figure 2, 3, 5), further investigations are need on the mechanisms underlying QA and ICAB combination effects and the application in mastitis prevention and treatment.

## Conclusion

Our data showed that co-treatment of QA and ICAB had higher inhibitory effects on LPS-induced inflammation and pyroptosis in MAC-T cells and mouse mammary glands than their individual treatment. Furthermore, combination of QA and ICAB effectively inhibited the activation of NF-κB and NLRP3 pathways compared to QA or ICAB treatment alone. Our data provide novel insights into the development of a promising therapy for mastitis in dairy cows.

## Data Availability

The original contributions presented in the study are included in the article/supplementary material, further inquiries can be directed to the corresponding authors.
